# Centerpiece plate vs. Arch plate fixation in cervical unilateral open-door laminoplasty: a retrospective comparative study

**DOI:** 10.3389/fsurg.2025.1482974

**Published:** 2025-02-06

**Authors:** Lishuang Huo, Fengyu Liu, Xianze Sun

**Affiliations:** ^1^ Department of Endocrinology, Shijiazhuang People’s Hospital, Shijiazhuang, China; ^2^ Department of Spine Surgery, The Third Hospital of Shijiazhang, Shijiazhuang, China

**Keywords:** Centerpiece plate, Arch plate, cervical, open-door, laminoplasty

## Abstract

**Purpose:**

To compare the clinical and radiological outcomes of Centerpiece plate and Arch plate fixation in cervical unilateral open-door laminoplasty.

**Methods:**

This study included 102 patients who underwent cervical unilateral open-door laminoplasty with Centerpiece plate fixation (62 patients) or Arch plate fixation (40 patients) between September 2017 and September 2022. Clinical and radiological outcomes were evaluated.

**Results:**

There were no significant differences in operation time, blood loss, and lamina open angle between the two groups. Before surgery, the two groups had comparable Japanese Orthopedic Association (JOA) scores and Pavlov's ratios. After surgery, the spinal drift distance and Pavlov ratio of the Centerpiece group were smaller than those of the Arch group. Both groups showed significant improvements in JOA scores after surgery and at the last follow-up compared to pre-surgery. At the final follow-up, the Centerpiece group's JOA scores and JOA score improvement rate were lower than those of the Arch group.

**Conclusions:**

Both Centerpiece plate and Arch plate fixation can improve the patient's symptoms. Centerpiece plate fixation has a worse prognosis than Arch plate fixation in cervical unilateral open-door laminoplasty because the ventral prong in the Centerpiece plate may obstruct the spinal cord's backward movement.

## Introduction

Hirabayashi first described cervical unilateral open-door laminoplasty in 1978 as a treatment for cervical myelopathy caused by cervical canal stenosis, spondylosis, and posterior longitudinal ligament ossification (OPLL) (1). The procedure was claimed to be effective in achieving neurologic recovery by expanding the spinal canal and providing enough space for the spinal cord to move away from the anterior compression (2–4). In comparison to cervical laminectomy, cervical unilateral open-door laminoplasty may preserve the cervical spine's dorsal parts while avoiding issues such as kyphosis and iatrogenic instability (5). Compared with laminectomy and fusion, laminoplasty has the benefits of shorter operative time time, less estimated blood loss, and reduced incidence of C5 palsy as well as overall complication rate (6). Compared with anterior decompression and fusion, laminoplasty showed similar results in terms of neurological recovery but was associated with a lower incidence of surgical complications (7).

Cervical laminoplasty requires a sufficient open angle and expansion of the spinal canal (8). After the laminae were opened, preventing restenosis was the main issue (9). Traditionally, the laminar door was held open using stay sutures put through the spinous process, the facet capsule, and the paravertebral muscle on the hinge side (5). However, it may cause subsequent constriction of the spinal canal as the lamina re-closes during long-term follow-up (10, 11). Moreover, titanium plate is preferable to suture suspensory with more range of motion and lower incidence of axial symptoms and C5 paralysis (12).

**Figure 1:**
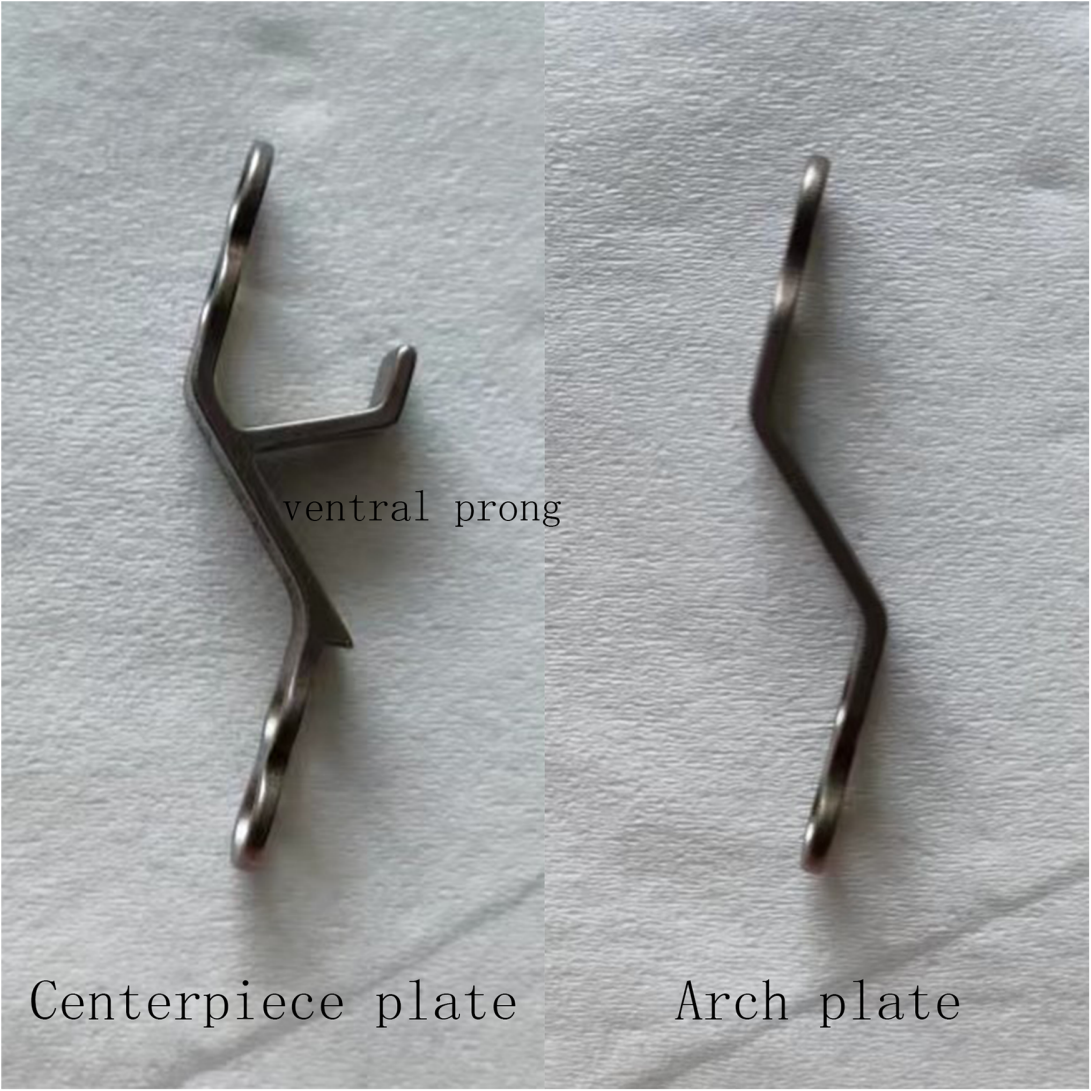
The centerpiece plate has two ventral prongs, while the arch plate does not.

The Arch mini-plate fixation technique, first published by O'Brien in 1996, was meant to keep the lamina open in a stable way by providing the laminae with instant hard support (11). Despite its effectiveness and appeal, the Arch mini-plate fixation technique is technically challenging. The surgeon and assistant must work together to keep the lamina and plate in position while drilling, tapping, and inserting the necessary screws (9). In 2004, Andrew E Park created the Centerpiece mini-plate mounting device to expedite the technique (9). With the help of two ventral prongs, the design provides an initial barrier against displacement of either the plate or the laminate (Figure 1).

However, the ventral prong of Centerpiece plate had been expanded into the spinal canal. Whether it will cause compression on the dorsal side of the spinal cord? Does the ventral prong impede the spinal cord's backward drift, affecting the clinical success of cervical unilateral open-door laminoplasty? To date, no comparison studies comparing Centerpiece plate and Arch plate fixation have been reported. The aim of this study was to compare the clinical and radiological effects of Centerpiece plate and Arch plate fixation in cervical unilateral open-door laminoplasty.

## Materials and methods

This retrospective comparison study was approved by the Institutional Ethics Committee. All patients provided written informed consent.

This study included patients who underwent cervical unilateral open-door laminoplasty with Centerpiece plate fixation or Arch plate fixation between September 2017 and September 2022. Inclusion criteria were as follows: (1) Cervical spondylotic myelopathy (CSM) or ossification of the posterior longitudinal ligament (OPLL) was diagnosed; (2) cervical unilateral open-door laminoplasty was performed; and (3) Centerpiece plate or Arch plate fixation was used. The exclusion criteria were as follows: (1) structural spinal deformity, (2) fractures, tumors, and metabolic problems, (3) prior cervical spine surgery, and (4) concurrent anterior cervical spine treatments.

### Surgical procedures

Following general anesthesia, the patient is put into a prone posture. The Mayfield device is put in place to stabilize the head and neck, preferably in a slight flexion. A conventional posterior midline exposure is used, and the paravertebral muscles are retracted laterally. All bilateral muscles connected to the C2 spinous processes are maintained intact. To preserve the integrity of the C2 spinous processes, patients with C2/3 stenosis are treated with a dome-like laminoplasty. For patients with C6/C7 stenosis, a partial laminectomy of C7 was performed to protect the integrity of the C7 spinous processes. The side with the most severe symptoms is designated as the open side, while the opposite side serves as the hinged side. Unilateral open-door laminoplasty is performed at the C3-C6 vertebrae. Centerpiece or Arch plates (Double Medical, China) are chosen based on the surgeons' preference. Two 8-mm screws are used to anchor the plate to the lateral mass, while two 6-mm screws are used to anchor the plate to the laminae. All patients are required to wear rigid cervical collars for four weeks after surgery.

### Clinical evaluation

Visual Analogue Scale (VAS) of axial neck pain was obtained prior to surgery and at the final follow-up. The Japanese Orthopedic Association (JOA) scores were obtained prior to surgery, one month following surgery, and at the final follow-up. The neurological recovery rate was calculated as (postoperative JOA score−preoperative score)/(17-preoperative score) × 100%. Complications were recorded during the intraoperative period and during postoperative follow-up.

### Radiologic evaluation

**Figure 2:**
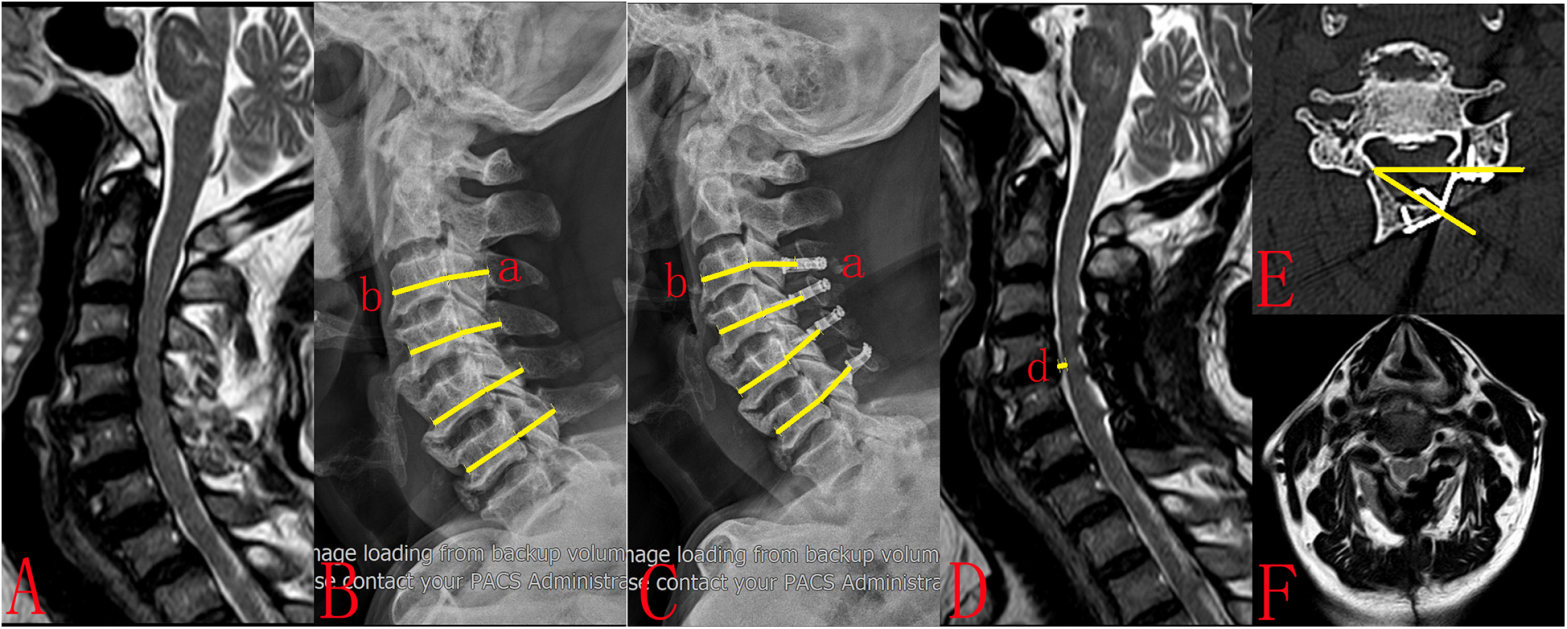
A 67-year-old female patient had been experiencing upper-limb numbness and asthenia for 10 months. (**A**) Preoperative MRI showed multisegmental spinal cord compression. (**B**) Preoperative plain radiograph showed cervical canal stenosis (Pavlov ratio = 0.74). (**C**) The patient underwent cervical unilateral open-door laminoplasty. Centerpiece plates were utilized at the C3-C6 segments (Pavlov ratio = 0.86). (**D**) Postoperative MRI demonstrated substantial posterior spinal cord drift (*d* = 2.73 mm). (**E**) Postoperative CT showed that the lamina open angle at C5 was 32.31. However, the ventral prong did not completely adhere to the lamina and instead expanded into the spinal canal. (**F**) Postoperative MRI demonstrated that the ventral prong in the Centerpiece plate obstruct the spinal cord's backward movement. The ventral prong of Centerpiece plate caused compression on the dorsal side of the spinal cord (**D,F**).

**Figure 3:**
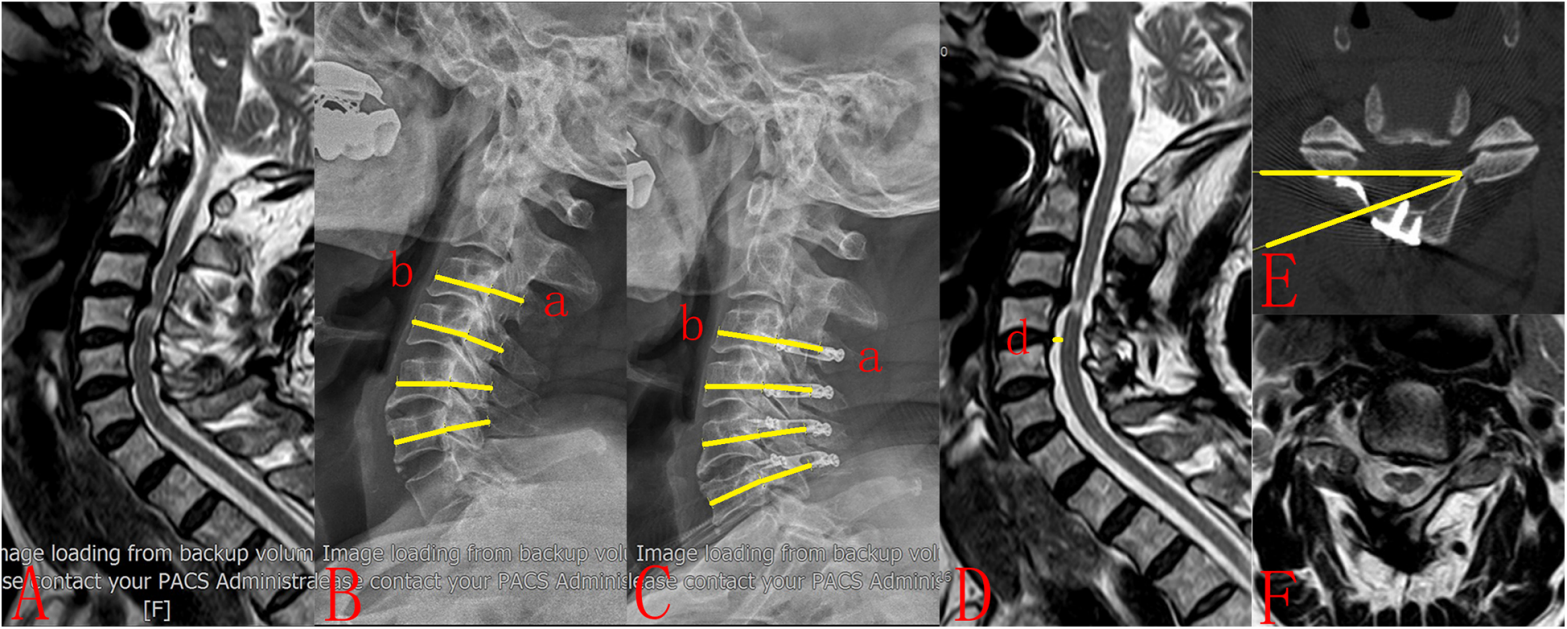
A 62-year-old male patient had been experiencing numbness and asthenia in his limbs for 1 year. (**A**) Preoperative MRI showed multisegmental spinal cord compression. (**B**) Preoperative plain radiograph showed cervical canal stenosis (Pavlov ratio = 0.73). (**C**) The patient underwent cervical unilateral open-door laminoplasty. Centerpiece plates were utilized at the C3-C6 segments (Pavlov ratio = 0.84). (**D**) Postoperative MRI demonstrated substantial posterior spinal cord drift (*d* = 3.26 mm). (**E**) Postoperative CT showed that the lamina open angle at C3 was 20.72. (**F**) Postoperative MRI demonstrated that the spinal cord was completely decompressed.

All patients underwent x-rays, CT scans, and MRIs before and after surgery. The Pavlov ratio was assessed preoperatively and postoperatively at C3–C6 on lateral x-ray radiographs. The sagittal diameter of the spinal canal (a) is measured from the posterior surface of the vertebral body to the nearest point on the corresponding spinal laminar line. The sagittal diameter of the vertebral body (b) is measured at the midpoint of the anterior and posterior surfaces. The Pavlov ratio is calculated using the formula a/b (13). Notably, in Centerpiece plate group, the spinal canal (a) is defined as the distance between the posterior surface of the vertebral body and the ventral prong (Figure 2). The open angle was measured for C3-C6 vertebra using CT scans taken one week after surgery (Figure 3). This angle was defined as the angle between the posterior line of the vertebral body and the line connecting the edges of the opened lamina on the axial image (14, 15). The distances between the posterior margin of the C4/5 vertebral disc and the nearest point of the anterior margin of the spinal cord were measured on pre- and postoperative T2-weighted mid-sagittal magnetic resonance imaging. The spinal drift distance at the C4/5 level equals the postoperative distance minus the preoperative distance (16).

### Statistical analysis

Data were entered into SPSS (version 21.0; SPSS Inc., Chicago, Illinois, USA) and assessed for normality using the Shapiro–Wilk test. Normal distribution values were presented as means ± standard deviation and compared between the two groups using independent t test. Non-normal distribution values were expressed as medians and interquartile ranges and compared between the two groups using the Mann–Whitney *U* test. The chi-squared test was employed to compare categorical variables. *P*-values <0.05 were considered statistically significant.

## Results

### Patient characteristics

Of the 118 patient records reviewed for potential inclusion, 16 were excluded for various reasons: incomplete or absent follow-up records (*n* = 9), nonadherence to C3-C6 decompression laminoplasty protocol (*n* = 3), history of prior cervical surgeries (*n* = 2), and the employment of combined anterior and posterior open-door laminoplasty (*n* = 2). This study comprised 102 patients that fit the criteria. Centerpiece group consisted of 62 patients who had Centerpiece plate fixation, with 47 males and 15 females. The average age was 63.94 ± 6.38 years. The 40 patients who underwent Arch plate fixation were classified into Arch group, which included 31 males and 9 females with a mean age of 64.80 ± 6.92. The mean follow-up period for the two groups was 29.47 ± 8.33 months and 30.28 ± 8.9 months, respectively. The duration of symptoms for two groups was 15.92 ± 7.71 months and 17.70 ± 9.75 months, respectively. The operating times for two groups were 149.03 ± 32.27 min and 161.50 ± 32.78 min, respectively. Blood loss for two groups was 247.58 ± 76.52 ml and 276.25 ± 82.42 ml, respectively. There were no significant variations in gender, age, symptom duration, follow-up time, operation time, or blood loss between the two groups (Table 1).

**Table 1:** Patient characteristics.

Characteristics	Centerpiece plate	Arch plate	*P*
Number of patients	62	40	
Gender (male: female)	47:15	31:9	0.844
Age (years)	63.94 ± 6.38	64.80 ± 6.92	0.520
Diagnose (CSM:OPLL)	49:13	33:7	0.667
Duration of symptom (months)	15.92 ± 7.71	17.70 ± 9.75	0.308
Operation time (minutes)	149.03 ± 32.27	161.50 ± 32.78	0.061
Blood loss (ml)	247.58 ± 76.52	276.25 ± 82.42	0.076
Size of plates (*n*, %)			0.230
8 mm	23 (9.3)	10 (6.3)	
10 mm	160 (64.5)	97 (60.6)	
12 mm	65 (26.2)	53 (33.1)	
Follow-up (month)	29.47 ± 8.33	30.28 ± 8.9	0.643
Complications			0.473
C5 palsy	0	1	
Axial pain	5	4	
Cerebrospinal fluid leakage	1	1	

CSM, cervical spondylosis myelopathy; OPLL, ossification of the posterior longitudinal ligament.

### Clinical results

The mean VAS dropped from 2.18 ± 1.08 preoperatively to 1.42 ± 0.84 at the final follow-up in Centerpiece plate group (*P* < 0.05) and from 2.35 ± 1.05 preoperatively to 1.58 ± 0.84 at the final follow-up in Arch plate group (*P* < 0.05). There was no significant difference in VAS between two groups.

In the Centerpiece group, the mean JOA score improved from 8.44 ± 2.27 preoperatively to 11.71 ± 2.32 (*P* < 0.001) one month after surgery and 13.79 ± 1.41 (*P* < 0.001) at the final follow-up. In the Arch group, the mean JOA score improved from 8.60 ± 2.15 preoperatively to 12.38 ± 2.07 (*P* < 0.001) one month after surgery and 14.48 ± 1.09 (*P* < 0.001) at the final follow-up. There were no significant differences in JOA scores between the two groups preoperatively or one month after surgery. At the final follow-up, the Centerpiece group's JOA scores and JOA score improvement rate were lower than those of the Arch group (*P* < 0.001).

### Radiologic results

In the Centerpiece group, the Pavlov ratio improved from 0.71 ± 0.04 preoperatively to 0.84 ± 0.05 postoperatively. In the Arch group, the Pavlov ratio increased from 0.72 ± 0.04 preoperatively to 1.00 ± 0.05 postoperatively. There was no significant difference in preoperative Pavlov's ratio between the two groups. However, the Pavlov ratio in the Centerpiece group was lower than the Arch group at postoperative (*P* < 0.001). The spinal drift distance in the Centerpiece group was lower than the Arch group at postoperative (*P* < 0.001). There were no significant difference in open angle between the two groups postoperatively (Table 2).

**Table 2:** Comparison of radiologic and clinical outcomes between the two groups.

	Centerpiece plate	Arch plate	*P*
Preoperative			
Pavlov ratio	0.71 ± 0.04	0.72 ± 0.04	0.318
VAS score	2.18 ± 1.08	2.35 ± 1.05	0.428
JOA score	8.44 ± 2.27	8.60 ± 2.15	0.716
Postoperative			
Pavlov ratio	0.84 ± 0.05*	1.00 ± 0.05*	<0.001
Open angle (°)	34.76 ± 2.40	35.30 ± 2.44	0.271
JOA score	11.71 ± 2.32*	12.38 ± 2.07*	0.144
Spinal drift distance (mm)	2.17 ± 0.43	2.53 ± 0.36	<0.001
Final follow-up			
VAS score	1.42 ± 0.84*	1.58 ± 0.84*	0.364
JOA score	13.79 ± 1.41*	14.48 ± 1.09*	0.011
JOA recovery rate (%)	63.32 ± 11.26	69.87 ± 11.14	<0.001

VAS, Visual Analogue Scale; JOA, Japanese Orthopedic Association.

* *P* < 0.001 compared with the preoperative parameter.

### Complications

One patient reported C5 palsy (in the Arch group). The patient fully healed after three months of conservative treatment. Nine individuals reported axial pain (5 in the Centerpiece group and 4 in the Arch group). All patients were pain-free within three months after starting oral analgesics. Two individuals had cerebrospinal fluid leaking (1 in the Centerpiece group and one in the Arch group). The drainage tube was removed seven days after the operation, and the incision was sutured. Both patients healed without complications. The incidences of complications did not differ significantly between the two groups.

## Discussion

Mini-plates are extensively utilized in cervical unilateral open-door laminoplasty (14, 17). They can be used to stabilize the lamina and avoid re-closing the door. The two most typically utilized plates are arch plates and centerpiece plates (2, 3, 8–11). The choice of the two plates depends on the surgeons' preference. To date, no research comparing two plates have been published. The aim of this study was to compare the clinical and radiological effects of Centerpiece plate and Arch plate fixation in cervical unilateral open-door laminoplasty.

This study included 102 patients who underwent cervical unilateral open-door laminoplasty with Centerpiece plate fixation (62 patients) or Arch plate fixation (40 patients). Both groups showed significant improvements in JOA scores after surgery and at the last follow-up compared to pre-surgery.

Both Centerpiece plate and Arch plate fixation can improve the patient's symptoms. Because the spinal cord is severely compressed, mild decompression may alleviate the patient's symptoms.

According to Xu T et al, the overall complication rate of cervical laminoplasty was 14.3% (5 of 35), the mean VAS dropped from 4 (3–6) preoperatively to 2 (1–4) at the final follow-up, the mean JOA score improved from 10 (7–13) preoperatively to 15 (14–16) at the final follow-up (18). According to Tamai K et al, the overall complication rate of cervical laminoplasty was 10.7% (8 of 75), the mean VAS dropped from 25.1 ± 29.5 mm preoperatively to 14.4 ± 18.3 mm two years after surgery, the mean JOA score improved from 9.8 ± 2.7 preoperatively to 14.0 ± 2.0 two years after surgery (19). The data presented in these literature are similar to our research findings.

The study examined the clinical and radiological effects of two mini-plates. There were no significant differences in operation time, blood loss, and lamina open angle between the two groups. Before surgery, the two groups had comparable JOA scores and Pavlov's ratios. After surgery, the spinal drift distance and Pavlov ratio of the Centerpiece group were smaller than those of the Arch group. At the final follow-up, the Centerpiece group's JOA scores and JOA score improvement rate were lower than those of the Arch group. As a result, Centerpiece plate fixation has a worse prognosis than Arch plate fixation in cervical unilateral open-door laminoplasty. The main explanation could be that the ventral prong in the Centerpiece plate obstructs the spinal cord's backward movement. Thus, complete decompression cannot be achieved.

In this study, in Centerpiece plate group, the spinal canal (a) is defined as the distance between the posterior surface of the vertebral body and the ventral prong. In Arch plate group, the sagittal diameter of the spinal canal (a) is measured from the posterior surface of the vertebral body to the nearest point on the corresponding spinal laminar line. As the ventral prong expanding into the spinal canal, the sagittal diameter of the spinal canal (a) of Centerpiece plate group is smaller than that of Arch group, Pavlov ratio of Centerpiece group is smaller than that of Arch group.

In this study, there were no significant differences in lamina open angle between the two groups. With the same lamina open angle, the deeper the ventral prong extended into the spinal canal, the smaller the volume of the spinal canal. If the ventral prong is not completely adhere to the lamina and instead extend into the spinal canal, it will cause compression on the dorsal side of the spinal cord. As the ventral prong in the Centerpiece plate obstructs the spinal cord's backward movement, the spinal drift distance of the Centerpiece group is smaller than that of the Arch group.

Because the spinal cord was severely compressed prior to surgery, the Pavlov ratio of the two groups improved following the cervical unilateral open-door laminoplasty, resulting in improved clinical symptoms. However, because the Centerpiece plate group's postoperative Pavlov ratio was lower than that of the Arch group, the spinal drift distance was lower as well, resulting in a lower JOA recovery rate in the Centerpiece plate group than in the Arch group.

The cervical spinal canal is a closed ring-shaped structure that contains the spinal cord. The purpose of cervical unilateral open-door laminoplasty is to achieve decompression by expanding the spinal canal and allowing the spinal cord to drift posteriorly. When the Centerpiece plate is applied, the ventral prong enters the spinal canal. If the ventral prong is closely attached to the lamina, it may not cause spinal cord compression. If the ventral prong is not closely attached to the lamina and enters the spinal canal too much, it will cause new compression on the spinal cord, resulting in poor prognosis. To avoid this situation from occurring, the following measures should be done. First, large-sized plates are selected to maximize the lamina open angle. Second, the titanium plate can be shaped so that the ventral prong is attached to the vertebral lamina. Third, intraoperative radiography allows for a check of the ventral prong. Hook can also be used to identify the ventral prong. If the ventral prong is not properly attached to the vertebral lamina, the necessary corrections should be made immediately. Fourth, titanium plate designs should be improved by shortening the ventral prong to prevent excessive penetration into the spinal canal. Due to the excessive entry of the ventral prong into the spinal canal, it causes new compression on the spinal cord. Therefore, we can improve the design the Centerpiece plate, design ventral prong of different sizes, and the surgeon can choose the Centerpiece plate according to the patient's condition. So that the ventral prong can close to the lamina, thus avoiding the compression of the spinal cord.

Some limitations should be highlighted. First, these procedures were conducted by four surgeons, two of whom chose to use Centerpiece plates and the other two preferred to use Arch plates, resulting in selection bias. Because this study is a retrospective single-institution analysis, randomized controlled trials with long-term follow-up are required to confirm these findings. In addition, this could be due to a design flaw in the titanium plate or a surgeon's lack of surgical ability. However, we believe this is not an accidental problem and should be of concern to spine surgeons.

## Conclusion

Both Centerpiece plate and Arch plate fixation can improve the patient's symptoms. Centerpiece plate fixation has a worse prognosis than Arch plate fixation in cervical unilateral open-door laminoplasty because the ventral prong in the Centerpiece plate may obstruct the spinal cord's backward movement.

## Data Availability

The original contributions presented in the study are included in the article/Supplementary Material, further inquiries can be directed to the corresponding author.
